# Systematic Calibration for Ultra-High Accuracy Inertial Measurement Units

**DOI:** 10.3390/s16060940

**Published:** 2016-06-22

**Authors:** Qingzhong Cai, Gongliu Yang, Ningfang Song, Yiliang Liu

**Affiliations:** 1 School of Instrument Science and Opto-Electronics Engineering, Beihang University, Beijing 100191, China; bhu17-yang@139.com (G.Y.); songnf@263.net (N.S.); 2 Space Star Technology Co., Ltd., Beijing 100101, China; yiliang_liu91@163.com

**Keywords:** systematic calibration, ultrahigh-accuracy inertial measurement unit, error modelling, Kalman filter

## Abstract

An inertial navigation system (INS) has been widely used in challenging GPS environments. With the rapid development of modern physics, an atomic gyroscope will come into use in the near future with a predicted accuracy of 5 × 10^−6^°/h or better. However, existing calibration methods and devices can not satisfy the accuracy requirements of future ultra-high accuracy inertial sensors. In this paper, an improved calibration model is established by introducing gyro g-sensitivity errors, accelerometer cross-coupling errors and lever arm errors. A systematic calibration method is proposed based on a 51-state Kalman filter and smoother. Simulation results show that the proposed calibration method can realize the estimation of all the parameters using a common dual-axis turntable. Laboratory and sailing tests prove that the position accuracy in a five-day inertial navigation can be improved about 8% by the proposed calibration method. The accuracy can be improved at least 20% when the position accuracy of the atomic gyro INS can reach a level of 0.1 nautical miles/5 d. Compared with the existing calibration methods, the proposed method, with more error sources and high order small error parameters calibrated for ultra-high accuracy inertial measurement units (IMUs) using common turntables, has a great application potential in future atomic gyro INSs.

## 1. Introduction

An inertial navigation system (INS) is widely used in military and civilian application domains because it is entirely self-contained and can provide high-rate position, velocity and attitude information. In the past few years, the kinds of new type of inertial sensors are invented based on various principles (optic, micro-electro-mechanical, and so on). Consequently, the inertial navigation technology for using different types of new inertial sensors are discussed and improved continuously. The INS using ultra-high accuracy inertial measurement units (IMUs) is a hot issue with the development of an atomic gyro.

With the rapid development of modern physics, atomic gyroscopes have been demonstrated in recent years [1]. More and more countries and organizations carried out the research on atomic gyros and achieved many milestones [2,3,4,5]. It is predicted that the accuracy of an atomic gyro will be better than 5 × 10^−6^°/h [6].

In the future, the atomic gyro will bring a revolutionary change to inertial navigation technology. Most of the techniques in traditional inertial navigation need to be improved or replaced in atomic gyro navigation. The calibration of IMUs is one of the key techniques, which affects the INS accuracy directly. If the traditional calibration methods are used in ultra-high accuracy IMUs, the calibration error will be much bigger than the errors caused by the inertial sensors themselves and thereby became the main error. The advantage of the high accuracy of atomic inertial sensors cannot be played by traditional calculation methods. Thus, high accuracy calibration is a key technique for the development of an ultra-high accuracy INS using atomic inertial sensors.

The accuracy of IMU calibration is restricted by two factors. One is the accuracy of the calibration model. For traditional systems, the linear simplified model is used in most calibration methods. For improving the calibration accuracy of an optic gyro IMU, Cai *et al.* [7] and Pan *et al.* [8] proposed different calibration methods while considering the accelerometer second order nonlinear scale factor. However, it cannot satisfy the requirement of ultra-high accuracy atomic gyro IMUs. For example, the effect of gravity on atoms in an atom interferometer is much bigger than that on photons in an optic interferometer, the g-sensitivity errors, which is also discussed by Chen *et al.* [9] and Zheng *et al.* [10], cannot be ignored in an atomic gyro IMU. Thus, it is necessary to introduce more error sources and high order small errors into the calibration model according to the characteristics of the future atomic gyro IMU. In this paper, an improved calibration model is established by introducing gyro g-sensitivity errors, accelerometer cross-coupling errors, and lever arm errors.

Another restriction factor is the accuracy of the calibration equipment and methods. In traditional IMU calibration methods, the IMU outputs are compared with known reference information obtained by specialized high-precision equipment, whose performance restricts the calibration accuracy. Even if the best three-axis turntable is used, the orientation control accuracy cannot be better than 1″, which cannot satisfy the accuracy requirements of ultra-high accuracy atomic gyro IMUs. Given the advantage of not requiring precise orientation controls, multi-position calibration methods and systematic calibration methods have been widely discussed for both low and high accuracy IMUs in recent years [7,11,12]. However, because more error terms are considered in the ultra-high accuracy calibration model, it cannot decouple all the parameters only by the norm information of gravity and the Earth’s rotation. The systematic calibration method proposed by Pan *et al.* [8] can solve all parameters relative to the gradient of velocity errors when the INS navigates in a given rotation sequence. However, in the ultra-high accuracy calibration model, the coupling relation between the parameters and the navigation errors are too complex to deduce the analytical solution of all parameters. In this paper, an optimal estimation smoother based on the complex calibration model is designed, and an improved rotation sequence is given for decoupling all of the parameters in the model.

This paper is organized as follows. In Section 2, the calibration model of an ultra-high accuracy IMU is established. In Section 3, the systematic calibration method based on an optimal estimation filter is designed. In Section 4, the calibration method is verified by simulation, laboratory and sailing tests. Finally, the conclusion is given in Section 5.

## 2. Calibration Model of Ultra-High Accuracy IMUs

### 2.1. Calibration Model of Ultrahigh-Accuracy Gyro Triads

**Figure 1:**
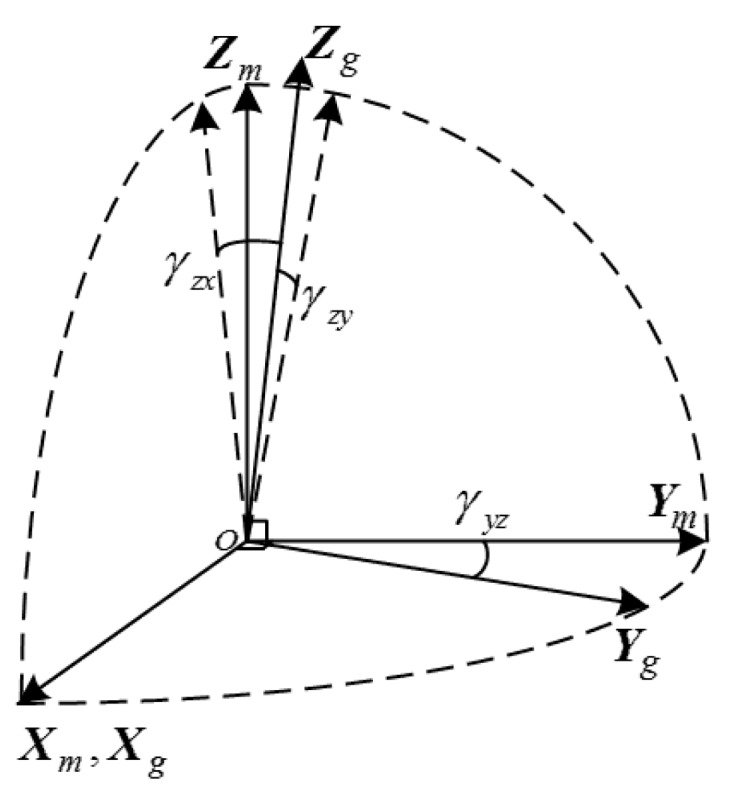
Definition of IMU frame.

An IMU for inertial navigation consists of three orthogonal gyros and three orthogonal accelerators. In order to clarify the physical meaning of misalignment, the gyro axes are selected as a base to establish the IMU frame (denoted by symbol *m*) to transforming all of the sensor outputs into an orthogonal coordinate frame [7]. First, the $X_{m}$ axis of the IMU frame is defined to be coincided with the $X_{g}$ gyro input axis, and then the $Y_{m}$ axis is defined by one small angle rotation from $Y_{g}$ in the $X_{g}Y_{g}$ plane, and the $Z_{m}$ axis is defined by one small angle rotation around the $X_{m}$ axis and another small rotation around the $Y_{m}$ axis (shown in Figure 1). Therefore, the misalignment of gyro can be represented by three small angles $\gamma_{yz}$, $\gamma_{zx}$ and $\gamma_{zy}$ in the IMU frame.

Taking the *x*-gyro, for example, the gyro measurement equation, which takes g-sensitivity errors into account, can be represented as:

(1) $$K_{gx}N_{gx}=\omega_{x}+b_{gx}+v_{gx}+G_{xx}f_{x}+G_{xy}f_{y}+G_{xz}f_{z}$$

where $K_{gx}$ is the scale factor of the *x*-gyro; $N_{gx}$ is the *x*-gyro output before compensation; $\omega_{x}$ is the true angle velocity in the *x*-axis direction; $b_{gx}$ and $v_{gx}$ are the bias and measurement noise of the *x*-gyro; $G_{xx}$, $G_{xy}$, $G_{xz}$ are the g-sensitivity coefficients of the x-gyro in the *x*-, *y*- and *z*-axis directions; and $f_{x}$, $f_{y}$, $f_{z}$ are the true accelerations in *x*-, *y*- and *z*-axis directions. If the *z*-axis points upwards, then $f_{z}$ equals the gravitational acceleration *g*, and $G_{xz}$ plays the dominant role. Because the strapdown INS does not have a stable platform to trace the geographic frame, g-sensitivity coefficients in all directions should be calibrated.

Performing the transposition transform on Equation (1), we can get the calibration model of the *x*-gyro as:

(2) $${\tilde{\omega }}_{x}=K_{gx}N_{gx}-b_{gx}-v_{gx}-G_{xx}{\tilde{f}}_{x}-G_{xy}{\tilde{f}}_{y}-G_{xz}{\tilde{f}}_{z}$$

where ${\tilde{\omega }}_{x}$ is the measurement of angle velocity in the *x*-gyro direction after calibration and compensation, ${\tilde{f}}_{x}$, ${\tilde{f}}_{y}$, ${\tilde{f}}_{z}$ are the measurements of accelerations in *x*-, *y*- and *z*-axis directions.

The calibration model of gyro triad is defined in the *m*-frame in Figure 1. Ignoring the high order terms, the model can be expressed as:

(3) $${\tilde{\omega }}^{m}=T_{g}^{m}K_{g}N_{g}-b_{g}^{m}-v_{g}^{m}-G{\tilde{f}}^{m}$$

where ${\tilde{\omega }}^{m}$, $b_{g}^{m}$ and $v_{g}^{m}$ are the measurements, bias and noise vectors of the gyro triads in *m*-frame, ${\tilde{\omega }}^{m}={\left[\begin{array}{ccc} {\tilde{\omega }}_{x}^{m} & {\tilde{\omega }}_{y}^{m} & {\tilde{\omega }}_{z}^{m} \end{array}\right]}^{T}$, $b_{g}^{m}={\left[\begin{array}{ccc} b_{gx}^{m} & b_{gy}^{m} & b_{gz}^{m} \end{array}\right]}^{T}$, $v_{g}^{m}={\left[\begin{array}{ccc} v_{gx}^{m} & v_{gy}^{m} & v_{gz}^{m} \end{array}\right]}^{T}$; $K_{g}=\left[\begin{array}{ccc} K_{gx} & 0 & 0\\ 0 & K_{gy} & 0\\ 0 & 0 & K_{gz} \end{array}\right]$ is the scale factor matrix of the gyro triad; $N_{g}={\left[\begin{array}{ccc} N_{gx} & N_{gy} & N_{gz} \end{array}\right]}^{T}$ is the gyro output vector; $T_{g}^{m}\approx \left[\begin{array}{ccc} 1 & 0 & 0\\ -\gamma_{yz} & 1 & 0\\ \gamma_{zy} & -\gamma_{zx} & 1 \end{array}\right]$ is the misalignment matrix; $G=\left[\begin{array}{ccc} G_{xx} & G_{xy} & G_{xz}\\ G_{yx} & G_{yy} & G_{yz}\\ G_{zx} & G_{zy} & G_{zz} \end{array}\right]$ is the g-sensitivity coefficient matrix; and ${\tilde{f}}^{m}={\left[\begin{array}{ccc} {\tilde{f}}_{x}^{m} & {\tilde{f}}_{y}^{m} & {\tilde{f}}_{z}^{m} \end{array}\right]}^{T}$ is the acceleration vector measured by the accelerator in *m*-frame.

### 2.2. Calibration Model of Ultrahigh-Accuracy Accelerometer Triad

Taking the *x*-accelerator for example, the measurement equation of the *x*-accelerator considering a nonlinear scale factor and cross-coupling errors can be expressed as:

(4) $$K_{ax}N_{ax}=f_{x}+b_{ax}+v_{ax}+K_{axx}f_{x}^{2}+K_{axy}f_{x}f_{y}+K_{axz}f_{x}f_{z}$$

where $K_{ax}$ is the scale factor; $N_{ax}$ is the output before compensation; $f_{x}$ is the true accelerations in the *x*-axis direction; $b_{ax}$ and $v_{ax}$ are the bias and measurement noise; $K_{axx}$ is the nonlinear scale factor; and $K_{axy}$ and $K_{axz}$ are the cross-coupling errors of the *x*-accelerator in *y*- and *z*-axis directions.

Performing the transposition transform on Equation (4), we can get the calibration model of the *x*-accelerator as:

(5) $${\tilde{f}}_{x}=K_{ax}N_{ax}-b_{ax}-v_{ax}-K_{axx}{\tilde{f}}_{x}^{2}-K_{axy}{\tilde{f}}_{x}{\tilde{f}}_{y}-K_{axz}{\tilde{f}}_{x}{\tilde{f}}_{z}$$

where ${\tilde{f}}_{x}$, ${\tilde{f}}_{y}$, ${\tilde{f}}_{z}$ are the measurements of accelerations in *x*-, *y*- and *z*-axis directions after calibration and compensation.

The calibration model of accelerator triad is defined in the *m*-frame in Figure 1. Ignoring the high order terms, the model can be expressed as:

(6) $${\tilde{f}}^{m}=T_{a}^{m}K_{a}N_{a}-b_{a}^{m}-v_{a}^{m}-K_{a2}{\tilde{f}}^{m(2)}-K_{cross}{\tilde{f}}^{m(cross)}$$

where ${\tilde{f}}^{m}$, $b_{a}^{m}$ and $v_{a}^{m}$ are the measurements, bias and noise vectors in *m*-frame; $K_{a}$ and $N_{a}$ are the scale factor matrix and output vector; $T_{a}^{m}\approx \left[\begin{array}{ccc} 1 & \alpha_{xz} & -\alpha_{xy}\\ -\alpha_{yz} & 1 & \alpha_{yx}\\ \alpha_{zy} & -\alpha_{zx} & 1 \end{array}\right]$ is the misalignment matrix; $K_{a2}=\left[\begin{array}{ccc} K_{axx} & 0 & 0\\ 0 & K_{ayy} & 0\\ 0 & 0 & K_{azz} \end{array}\right]$ is the nonlinear scale factor matrix; ${\tilde{f}}^{m(2)}=[\begin{array}{ccc} {\left({\tilde{f}}_{x}^{m}\right)}^{2} & {\left({\tilde{f}}_{y}^{m}\right)}^{2} & {\left({\tilde{f}}_{z}^{m}\right)}^{2} \end{array}]$; $K_{cross}=\left[\begin{array}{ccc} K_{axy} & K_{axz} & 0\\ K_{ayx} & 0 & K_{ayz}\\ 0 & K_{azx} & K_{azy} \end{array}\right]$ is the cross-coupling scale factor matrix; and ${\tilde{f}}^{m(course)}=[\begin{array}{ccc} {\tilde{f}}_{x}^{m}{\tilde{f}}_{y}^{m} & {\tilde{f}}_{x}^{m}{\tilde{f}}_{z}^{m} & {\tilde{f}}_{y}^{m}{\tilde{f}}_{z}^{m} \end{array}]$.

In order to reduce the computation complexity for real-time solutions, the iterative method mentioned in [7] is used in this paper.

First, an initial approximation is calculated by the simplified linear model, which ignores the nonlinear scale factor and cross-coupling errors terms:

(7) $${\tilde{f}}_{\left(0\right)}^{m}=T_{a}^{m}K_{a}N_{a}-b_{a}^{m}$$

Then, the iteration (shown in Equation (8)) is kept to revise the correction of nonlinear scale factor term until the prospective precision is reached:

(8) $${\tilde{f}}_{\left(n\right)}^{m}={\tilde{f}}_{\left(n-1\right)}^{m}-K_{a2}{{\tilde{f}}_{\left(n-1\right)}^{m}}^{\left(2\right)}-K_{cross}{{\tilde{f}}_{\left(n-1\right)}^{m}}^{\left(cross\right)}$$

### 2.3. Calibration Model of Lever Arm Errors

In an ideal situation, the accelerator triad of the IMU in stapdown INS needs to be mounted exactly at the same position. Apparently, the ideal situation can not be achieved because the accelerator itself has a certain size, and the mounting position could be restricted as well. Due to the physical offset between the mounting position of the accelerator and the ideal measurement point of the IMU, the navigation errors will be generated by the tangential and centripetal force, which is caused by the vehicle’s angular movement and observed by the accelerator triad; this phenomenon is called the size effect or the lever arm effect.

**Figure 2:**
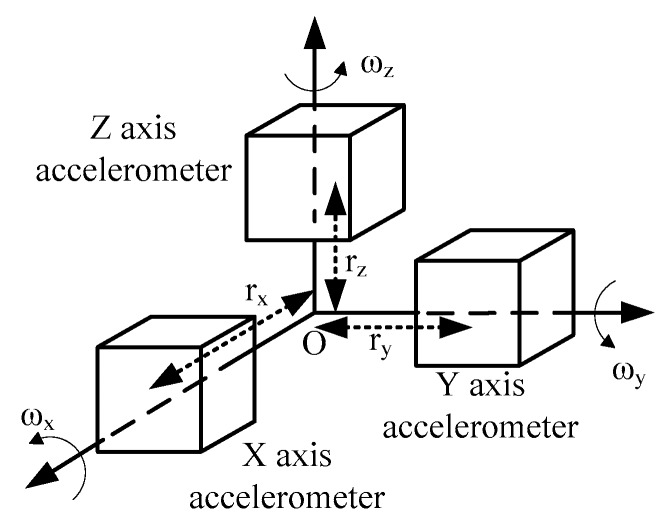
The sketch of size effect.

The principle of size effect is shown in Figure 2. The accelerator sensitive axes intersect at the measurement point of IMU (point O). The distance from the mounting position of the *x*-accelerator to the origin is *r_x_*, which is also called the lever arm error, and its sensitive axis points to the positive *x*-axis. If the system rotates around the *y*- and *z*-axis with the angle velocity of *ω_y_* and *ω_z_*, a centrifugal acceleration will be detected by the *x*-accelerator:

(9) $$a_{x}=-(\omega_{y}^{2}+\omega_{z}^{2})r_{x}$$

Similarly, the centrifugal accelerations detected by the *y*- and *z*-accelerator are:

(10) $$a_{y}=-(\omega_{x}^{2}+\omega_{z}^{2})r_{y}$$

(11) $$a_{z}=-(\omega_{x}^{2}+\omega_{y}^{2})r_{z}$$

The calibration model of accelerator triad can be further detailed as:

(12) $${\tilde{f}}^{m}=T_{a}^{m}K_{a}N_{a}-b_{a}^{m}-v_{a}^{m}-K_{2}{\tilde{f}}^{m(2)}-K_{cross}{\tilde{f}}^{m(cross)}-{\tilde{\omega }}^{m(size)}r$$

where ${\tilde{\omega }}^{m(size)}=\left[\begin{array}{ccc} 0 & {\left(\omega_{y}^{m}\right)}^{2} & {\left(\omega_{z}^{m}\right)}^{2}\\ {\left(\omega_{x}^{m}\right)}^{2} & 0 & {\left(\omega_{z}^{m}\right)}^{2}\\ {\left(\omega_{x}^{m}\right)}^{2} & {\left(\omega_{y}^{m}\right)}^{2} & 0 \end{array}\right]$, and $r={\left[\begin{array}{ccc} r_{x} & r_{y} & r_{z} \end{array}\right]}^{T}$ is the lever arm error vector.

## 3. Systematic Calibration Method Based on Optimal Estimation Filter

### 3.1. Principle of Systematic Calibration

**Figure 3:**
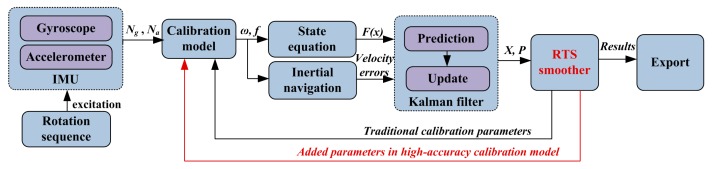
Integrated architecture of systematic calibration method.

The principle of the systematic calibration method proposed in this paper is shown in Figure 3. A 51-state Kalman filter is established by the INS error equation and the calibration model in Section 2. The Rauch-Tung-Striebel (RTS) smoothing method is used to improve the estimation accuracy off-line.

### 3.2. Kalman Filtering and RTS Smoothing

Considering that the error of the ultra-high accuracy IMU is quite small, the linearized model of the system is precise enough. Thus, a traditional external Kalman filter can realize a least variance estimation, while other complicated filters may decrease the estimation accuracy or increase the computation amount, and that is unnecessary for an already quite complicated state equation of systematic calibration. Based on the Kalman filter, smoothing the calibration parameters in the whole calibration process by RTS smoothing can further improve the estimation accuracy. The Kalman filter calculation steps can be found in [9].

The RTS smoothing is also called fixed-interval smoothing, which can estimate the states in the whole trajectory using discontinuous observable information. The calculation steps of RTS smoother are as below.

During the Kalman filter is working, the predicted states ${\hat{x}}_{k/k-1}$ and covariance $P_{k/k-1}$, the updated states ${\hat{x}}_{k}$ and covariance $P_{k}$ are all stored in memory for smoothing later, on the assumption that there are *M* times in the whole trajectory, and each time can be denoted as *j* (0<j<M). After the calculation of Kalman filter, the RTS smoother begins at time *M*. With j=M,M−1,⋯,1, the iterative equation of the state vector in the RTS smoother can be written as:

(13) $${\hat{x}}_{j-1/M}={\hat{x}}_{j-1}+A_{j-1}\left({\hat{x}}_{j/M}-{\hat{x}}_{j/j-1}\right)$$

(14) $$A_{j-1}=P_{j-1}\Phi_{j,j-1}^{T}P_{j/j-1}^{-1}$$

The iterative equation of the covariance matrix in the RTS smoother can be presented as:

(15) $$P_{j-1/M}=P_{j-1}+A_{j-1}\left(P_{j/M}-P_{j/j-1}\right)A_{j-1}^{T}$$

where the updated states and covariance at time *M* of the Kalman filter is the initial value of the RTS smoother:

(16) $${\hat{x}}_{M/M}={\hat{x}}_{M}$$

### 3.3. State Equation of Systematic Calibration Filter

In the navigation frame (E-N-U frame is chosen in this paper), the error equation of the INS can be written as:

(17) $$\begin{array}{c} \dot{\varphi }=\varphi \times \omega_{in}^{n}+\delta \omega_{in}^{n}-C_{b}^{n}\delta \omega_{ib}^{b}\\ \delta {\dot{V}}^{n}=f^{n}\times \varphi -(2\omega_{ie}^{n}+\omega_{en}^{n})\times \delta v^{n}+V^{n}\times (2\delta \omega_{ie}^{n}+\delta \omega_{en}^{n})+C_{b}^{n}\delta f^{b}-\delta g\\ \delta \dot{L}=\frac{\delta V_{N}}{R_{M}+h}-\frac{V_{N}\delta h}{{\left(R_{M}+h\right)}^{2}}\\ \delta \dot{\lambda }=\frac{\delta V_{E}}{\left(R_{N}+h\right)\cos L}+\frac{V_{E}\sin L\delta L}{\left(R_{N}+h\right)\cos^{2}L}-\frac{V_{E}\delta h}{{\left(R_{N}+h\right)}^{2}\cos L}\\ \delta \dot{h}=\delta v_{U} \end{array}\}$$

where $\varphi =[\begin{array}{ccc} \varphi_{E} & \varphi_{N} & \varphi_{U} \end{array}]$ is the attitude error angles, which are considered as small angles; $\omega_{in}^{n}$ is the rotation angle velocity of the navigation frame relative to the inertial frame, which is caused by the earth rotation and the vehicle movement; $\delta \omega_{in}^{n}$ is the estimation error of $\omega_{in}^{n}$ in the navigation solution; $f^{n}$ is the specific force in the navigation frame, $\omega_{ie}^{n}$ and $\omega_{en}^{n}$ are the angle velocity of the earth rotation and the angle velocity when the vehicle rotates around the earth, respectively; δg is the gravity vector error; $V^{n}={\left[\begin{array}{ccc} V_{E} & V_{N} & V_{U} \end{array}\right]}^{T}$ is the velocity relative to the earth; L, λ and h are the local latitude, longitude and height; $R_{M}$ and $R_{N}$ are the radii of the local earth meridian and prime vertical; $\delta \omega_{ib}^{b}$ and $\delta f^{b}$ are the measurement errors of the gyro and the accelerator.

In the systematic calibration, we defined the body frame (*b*-frame) as the IMU frame (*m*-frame) in Section 2.1, and the superscript *b* can be replaced by *m*. According to the simplified linear calibration model, the measurement errors of the gyro and the accelerator can be written as:

(18) $$\delta \omega^{m}=\delta K_{G}{\tilde{\omega }}^{m}-\delta b_{g}^{m}-\delta G{\tilde{f}}^{m}$$

(19) $$\delta f^{m}=\delta K_{A}{\tilde{f}}^{m}-\delta b_{a}^{m}-\delta K_{a2}{\tilde{f}}^{m(2)}-\delta K_{cross}{\tilde{f}}^{m(cross)}-{\tilde{\omega }}^{m(size)}\delta r$$

where $\delta b_{g}^{m}$ and $\delta b_{a}^{m}$ are the vectors of gyro and accelerometer bias errors; δG is the error vector of gyro g-sensitivity scale factor. $\delta K_{a2}$ and $\delta K_{cross}$ are the error vectors of the accelerometer nonlinear scale factor and the cross-coupling scale factor. δr is the error vector of lever arm error. $\delta K_{G}$ and $\delta K_{A}$are the scale factor and misalignment matrix of the gyro and the accelerator. Because the *m*-frame is defined by the gyro sensitive axes, $\delta K_{G}$ and $\delta K_{A}$ can be written as:

(20) $$\delta K_{A}=\delta T_{a}^{m}\delta K_{a}=\left[\begin{array}{ccc} \delta K_{ax} & \delta \alpha_{xz} & -\delta \alpha_{xy}\\ -\delta \alpha_{yz} & \delta K_{ay} & \delta \alpha_{yx}\\ \delta \alpha_{zy} & -\delta \alpha_{zx} & \delta K_{az} \end{array}\right]$$

Assuming that all the error terms to be calibrated are constants, then:

(21) $$\begin{array}{l} \delta {\dot{K}}_{G}=0_{3\times 3},\;\delta {\dot{K}}_{A}=0_{3\times 3},\;\delta {\dot{b}}_{a}^{m}=0_{1\times 3},\;\delta {\dot{b}}_{g}^{m}=0_{1\times 3}\\ \delta \dot{G}=0_{3\times 3},\;\delta {\dot{K}}_{a2}=0_{3\times 3},\;\delta {\dot{K}}_{cross}=0_{3\times 3},\;\delta \dot{r}=0_{1\times 3} \end{array}$$

According to the above error equation and calibration model of the INS, a 51-state Kalman filter is designed as:

(22) $$X^{51}={\left[\varphi^{T}\;\delta V^{T}\;\delta P^{T}\;X_{KG}^{T}\;{\left(\delta b_{g}^{m}\right)}^{T}\;X_{KA}^{T}\;{\left(\delta b_{a}^{m}\right)}^{T}\;X_{G}^{T}\;X_{Ka2}^{T}\;X_{Kcross}^{T}\;\delta r^{T}\right]}^{T}$$

where φ, δV and δP are the attitude error, velocity error and position error, respectively; $X_{\text{KG}}^{T}$, $X_{KA}^{T}$, $X_{G}^{T}$, $X_{Ka2}^{T}$ and $X_{Kcross}^{T}$ are the vector forms of $\delta K_{G}$, $\delta K_{A}$, δG, $\delta K_{a2}$ and $\delta K_{cross}$.

By the above Equations (17)–(19), the filter state equation can be expressed as:

(23) $$\dot{X}=FX+Gu$$

where

$$F=\left[\begin{array}{ccccccccc} F_{11} & F_{12} & F_{13} & F_{14} & 0_{3\times 12} & F_{16} & 0_{3\times 3} & 0_{3\times 6} & 0_{3\times 3}\\ F_{21} & F_{22} & F_{23} & 0_{3\times 9} & F_{25} & 0_{3\times 9} & F_{27} & F_{28} & F_{29}\\ 0_{3\times 3} & F_{32} & F_{33} & 0_{3\times 9} & 0_{3\times 12} & 0_{3\times 9} & 0_{3\times 3} & 0_{3\times 6} & 0_{3\times 3}\\ 0_{9\times 3} & 0_{9\times 3} & 0_{9\times 3} & 0_{9\times 9} & 0_{9\times 12} & 0_{9\times 9} & 0_{9\times 3} & 0_{9\times 6} & 0_{9\times 3}\\ 0_{33\times 3} & 0_{33\times 3} & 0_{33\times 3} & 0_{33\times 9} & 0_{33\times 12} & 0_{33\times 9} & 0_{33\times 3} & 0_{33\times 6} & 0_{33\times 3} \end{array}\right]$$

In the matrix ***F***:

$$F_{11}=\left[\begin{array}{ccc} 0 & \omega_{ie}\sin L+\frac{V_{E}}{R_{N}+h}\tan L & -\left(\omega_{ie}\cos L+\frac{V_{E}}{R_{N}+h}\right)\\ -\left(\omega_{ie}\sin L+\frac{V_{E}}{R_{N}+h}\tan L\right) & 0 & -\frac{V_{N}}{R_{M}+h}\\ \omega_{ie}\cos L+\frac{V_{E}}{R_{N}+h} & \frac{V_{N}}{R_{M}+h} & 0 \end{array}\right]$$

$$F_{12}=\left[\begin{array}{ccc} 0 & -\frac{1}{R_{M}+h} & 0\\ \frac{1}{R_{N}+h} & 0 & 0\\ \frac{\tan L}{R_{N}+h} & 0 & 0 \end{array}\right]$$

$$F_{13}=\left[\begin{array}{ccc} 0 & 0 & \frac{V_{N}}{{\left(R_{M}+h\right)}^{2}}\\ \omega_{ie}\sin L & 0 & -\frac{V_{E}}{{\left(R_{N}+h\right)}^{2}}\\ \omega_{ie}\cos L+\frac{V_{E}\sec^{2}L}{R_{N}+h} & 0 & -\frac{V_{E}\tan L}{{\left(R_{N}+h\right)}^{2}} \end{array}\right]$$

$$F_{14}=-C_{m}^{n}\left[\begin{array}{cccc} {\tilde{\omega }}_{imx}^{m}I_{3} & \left[\begin{array}{c} 0_{1\times 2}\\ {\tilde{\omega }}_{imy}^{m}I_{2} \end{array}\right] & \left[\begin{array}{c} 0_{2\times 1}\\ {\tilde{\omega }}_{imz}^{m} \end{array}\right] & I_{3} \end{array}\right]$$

$$F_{21}=\left[\begin{array}{ccc} 0 & -{\tilde{f}}_{U}^{n} & {\tilde{f}}_{N}^{n}\\ {\tilde{f}}_{U}^{n} & 0 & -{\tilde{f}}_{E}^{n}\\ -{\tilde{f}}_{N}^{n} & {\tilde{f}}_{E}^{n} & 0 \end{array}\right]$$

$$F_{16}=C_{m}^{n}\left[\begin{array}{ccc} {\left({\tilde{f}}^{m}\right)}^{T} & 0_{3\times 1} & 0_{3\times 1}\\ 0_{3\times 1} & {\left({\tilde{f}}^{m}\right)}^{T} & 0_{3\times 1}\\ 0_{3\times 1} & 0_{3\times 1} & {\left({\tilde{f}}^{m}\right)}^{T} \end{array}\right]$$

$$F_{22}=\left[\begin{array}{ccc} \frac{V_{N}\tan L-V_{U}}{R_{N}+h} & \left(2\omega_{ie}\sin L+\frac{V_{E}}{R_{N}+h}\tan L\right) & -\left(2\omega_{ie}\cos L+\frac{V_{E}}{R_{N}+h}\right)\\ -\left(2\omega_{ie}\sin L+\frac{V_{E}}{R_{N}+h}\tan L\right) & -\frac{V_{U}}{R_{M}+h} & -\frac{V_{N}}{R_{M}+h}\\ 2\left(\omega_{ie}\cos L+\frac{V_{E}}{R_{N}+h}\right) & \frac{2V_{N}}{R_{M}+h} & 0 \end{array}\right]$$

$$F_{23}=\left[\begin{array}{ccc} 2\omega_{ie}\left(V_{U}\sin L+V_{N}\cos L\right)+\frac{V_{E}V_{N}}{R_{N}+h}\sec^{2}L & 0 & \frac{V_{E}V_{U}-V_{E}V_{N}\tan L}{{\left(R_{N}+h\right)}^{2}}\\ -\left(2V_{E}\omega_{ie}\cos L+\frac{V_{E}^{2}}{R_{N}+h}\sec^{2}L\right) & 0 & \frac{V_{N}V_{U}}{{\left(R_{M}+h\right)}^{2}}+\frac{V_{E}^{2}\tan L}{{\left(R_{N}+h\right)}^{2}}\\ -2V_{E}\omega_{ie}\sin L & 0 & -\frac{V_{N}^{2}}{{\left(R_{M}+h\right)}^{2}}+\frac{V_{E}^{2}}{{\left(R_{N}+h\right)}^{2}} \end{array}\right]$$

$$F_{25}=C_{m}^{n}\left[\begin{array}{cccc} {\tilde{f}}_{x}^{m}I_{3} & {\tilde{f}}_{y}^{m}I_{3} & {\tilde{f}}_{z}^{m}I_{3} & I_{3} \end{array}\right]$$

$$F_{27}=C_{m}^{n}\left[\begin{array}{ccc} {\left({\tilde{f}}_{x}^{m}\right)}^{2} & 0 & 0\\ 0 & {\left({\tilde{f}}_{y}^{m}\right)}^{2} & 0\\ 0 & 0 & {\left({\tilde{f}}_{z}^{m}\right)}^{2} \end{array}\right]$$

$$F_{28}=C_{m}^{n}\left[\begin{array}{cccccc} {\tilde{f}}_{x}^{m}{\tilde{f}}_{y}^{m} & {\tilde{f}}_{x}^{m}{\tilde{f}}_{z}^{m} & 0 & 0 & 0 & 0\\ 0 & 0 & {\tilde{f}}_{x}^{m}{\tilde{f}}_{y}^{m} & {\tilde{f}}_{y}^{m}{\tilde{f}}_{z}^{m} & 0 & 0\\ 0 & 0 & 0 & 0 & {\tilde{f}}_{x}^{m}{\tilde{f}}_{z}^{m} & {\tilde{f}}_{y}^{m}{\tilde{f}}_{z}^{m} \end{array}\right]$$

$$F_{29}=C_{m}^{n}\left[\begin{array}{ccc} {\left({\tilde{\omega }}_{imy}^{m}\right)}^{2}+{\left({\tilde{\omega }}_{imz}^{m}\right)}^{2} & 0 & 0\\ 0 & {\left({\tilde{\omega }}_{imx}^{m}\right)}^{2}+{\left({\tilde{\omega }}_{imz}^{m}\right)}^{2} & 0\\ 0 & 0 & {\left({\tilde{\omega }}_{imx}^{m}\right)}^{2}+{\left({\tilde{\omega }}_{imy}^{m}\right)}^{2} \end{array}\right]$$

$$F_{32}=\left[\begin{array}{ccc} 0 & \frac{1}{R_{M}+h} & 0\\ \frac{\sec L}{R_{M}+h} & 0 & 0\\ 0 & 0 & 1 \end{array}\right]$$

$$F_{33}=\left[\begin{array}{ccc} 0 & 0 & -\frac{V_{N}}{{\left(R_{M}+h\right)}^{2}}\\ \frac{V_{E}\tan L\sec L}{R_{N}+h} & 0 & -\frac{V_{E}\sec L}{{\left(R_{N}+h\right)}^{2}}\\ 0 & 0 & 0 \end{array}\right]$$

The input of the filter is the measurement noise of the gyro and the accelerator $u={\left[\begin{array}{cc} u_{g}^{T} & u_{a}^{T} \end{array}\right]}^{T}$, the input matrix is:

(24) $$G=\left[\begin{array}{cc} -C_{m}^{n} & 0_{3\times 3}\\ 0_{3\times 3} & C_{m}^{n}\\ 0_{45\times 3} & 0_{45\times 3} \end{array}\right]$$

The observation equation of the filter is:

(25) $$Z=\left[{\tilde{V}}^{n}-V_{obv}\right]=HX+v$$

where ${\tilde{V}}^{n}$ is the velocity solution result of the INS, and v is the observation noise. The observation matrix is:

(26) $$H=\left[\begin{array}{ccc} 0_{3\times 3} & I_{3} & 0_{3\times 45} \end{array}\right]$$

The feedback compensation form of the filter estimation result is:

(27) $$\begin{array}{c} C_{m}^{n}=\left(I_{3}+\left[\varphi \times \right]\right){\tilde{C}}_{m}^{n}\\ V^{n}={\tilde{V}}^{n}-\delta V^{n}\\ L=\tilde{L}-\delta L,\lambda =\tilde{\lambda }-\delta \lambda ,h=\tilde{h}-\delta h\\ b_{a}^{m}={\tilde{b}}_{a}^{m}+\delta b_{a}^{m},b_{g}^{m}={\tilde{b}}_{g}^{m}+\delta b_{g}^{m}\\ \begin{array}{c} K_{G}={\tilde{K}}_{G}+\delta K_{G},K_{A}={\tilde{K}}_{A}+\delta K_{A}\\ K_{a2}={\tilde{K}}_{a2}+\delta K_{a2},K_{cross}={\tilde{K}}_{cross}+\delta K_{cross}\\ G=\tilde{G}+\delta G \end{array} \end{array}\}$$

### 3.4. Rotation Sequence

A systematic calibration path with a dual-axis turntable is designed to decouple all calibration parameters. Taking the U-T type turntable, for example, (the outer-axis is a U type with the rotation axis in the horizontal direction, the inner-axis is a T type and orthogonal to the outer-axis), the calibration path is shown in Table 1. This path has 18 times of rotation with a rotation velocity of 5 °/s; the whole rotation path is accomplished in 1 h with a pause of 180 s at each position. The former nine times of rotation (including twice 180° rotation in the single direction of each axis) is designed to stimulate the gyro scale factor error, the misalignment, and the accelerator lever arm error. The navigation errors caused by them are attitude errors in the rotation direction, attitude errors and velocity errors perpendicular to the rotation direction. Then, the attitude errors cause different velocity errors with the effect of gravity; the latter nine times of rotation (including the positions with each axis pointing to up, ground and horizon) is designed to stimulate the accelerator scale factor error, the misalignment, the second-order nonlinear error, the cross-coupling error, and the gyro g-sensitivity error. The navigation errors caused by them are velocity errors in the vertical direction, velocity errors perpendicular to the vertical direction, velocity errors with a square root of proportion of the gravity and attitude errors. These velocity errors have different forms. By matching the various forms of velocity errors to the propagation forms of states in the systematic error model, all of the parameters can be estimated, respectively, after all errors are stimulated by a round of rotation paths. In the actual calibration, the gyro random noise is very large, which leads to a relatively long time to estimate the gyro bias error in the Kalman filter. Thus, two or more times calibration rotation can be performed in one actual calibration to ensure the estimation curves of the calibrated parameters can be convergent.

## 4. Simulation and Laboratory Tests

### 4.1. Simulation Test

A group gyro and accelerometer data for calibration is generated according to the rotation path in Table 1. The calibration error is defined as: the scale factor error of both gyro and accelerator is 300 ppm; the misalignment of both gyro and accelerator is 180″, the gyro bias is 0.05°/h with a white noise of 0.00005°/h, the accelerator bias is 200 μg with a white noise of 1 μg; the g-sensitivity error of the gyro is 0.001°/h/g; the nonlinear scale factor error and the cross-coupling error of the accelerator are 300 μg/g^2^, the lever arm error of the accelerator is 2 cm; and the attitude error of the turntable is 1′ (1σ).

**Figure 4:**
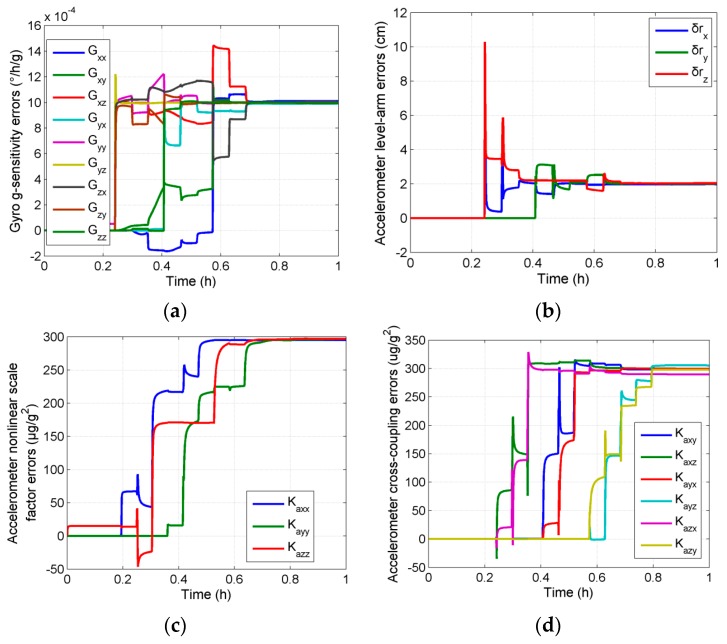
Estimation curves of the parameter errors in simulation tests (**a**) Gyro g-sensitivity errors; (**b**) Accelerometer level-arm errors; (**c**) Accelerometer nonlinear scale factor errors; (**d**) Accelerometer cross-coupling errors.

The calibration was performed using the proposed calibration method with the above data. The estimation curves of the parameter errors introduced in this paper are shown in Figure 4. All of the parameters got completely convergent in one group of calibration rotation. The errors of the parameters before and after filters are shown in Table 2. It can be seen that the self-calibration scheme proposed in this paper can make an effective estimation of all the parameters in the ultra-high precision calibration model.

### 4.2. Laboratory Test

**Figure 5:**
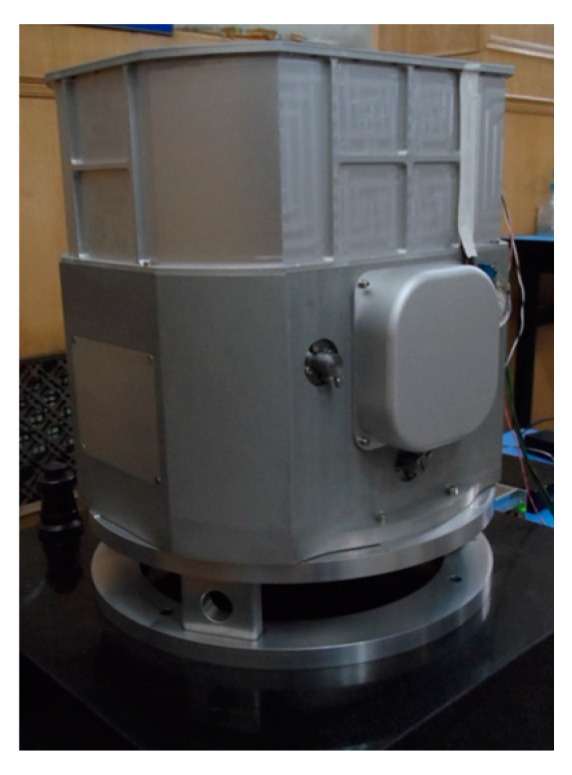
Marine dual-axis rotational INS.

A high accuracy marine dual-axis rotational INS (shown in Figure 5), whose accuracy is highest in existing INSs, is adopted to verify the proposed method. The 90-type ring laser gyros with the accuracy better than 0.003 °/h, and the quartz flexible accelerometers with an accuracy better than 10 μg are used in the IMU of the system. The sample frequency is 100 Hz.

**Figure 6:**
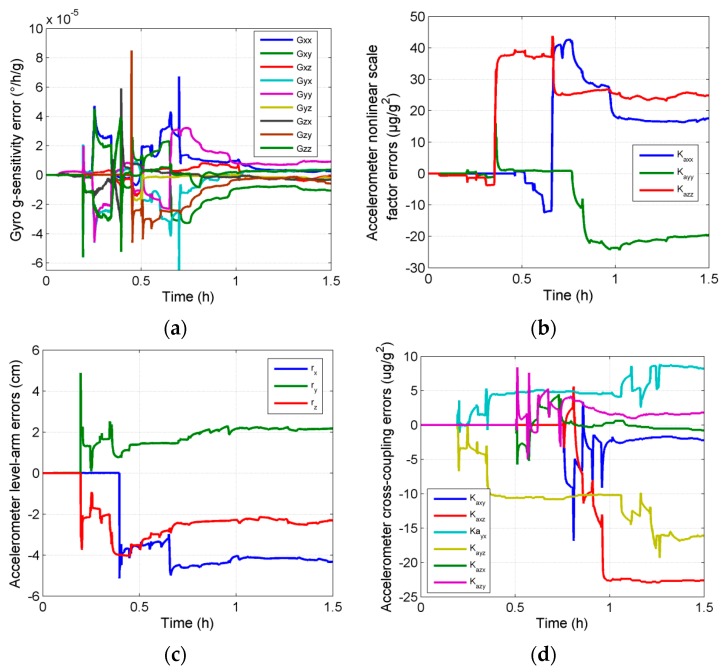
Estimation curves of the parameter errors in laboratory test (**a**) Gyro g-sensitivity errors; (**b**) Accelerometer level-arm errors; (**c**) Accelerometer nonlinear scale factor errors; (**d**) Accelerometer cross-coupling errors.

During the calibration, the system lays stably on a marble terrace in the laboratory as shown in Figure 6. The calibration rotation was performed using the dual-axis turntable of the dual-axis INS itself. The estimation curves of the parameter errors are shown in Figure 6. It can be seen that the convergence curves of the parameters caused by the bias instability and other random errors of the inertial sensors are fluctuant. For ultra-high accuracy IMUs, the sensors’ bias instability is small enough and is a close approximation to Gaussian white noise. Thus, it can rarely affect the accuracy of systematic calibration, and all parameters in the ultra-high accuracy calibration model can be estimated. The errors of the parameters are shown in Table 3.

**Figure 7:**
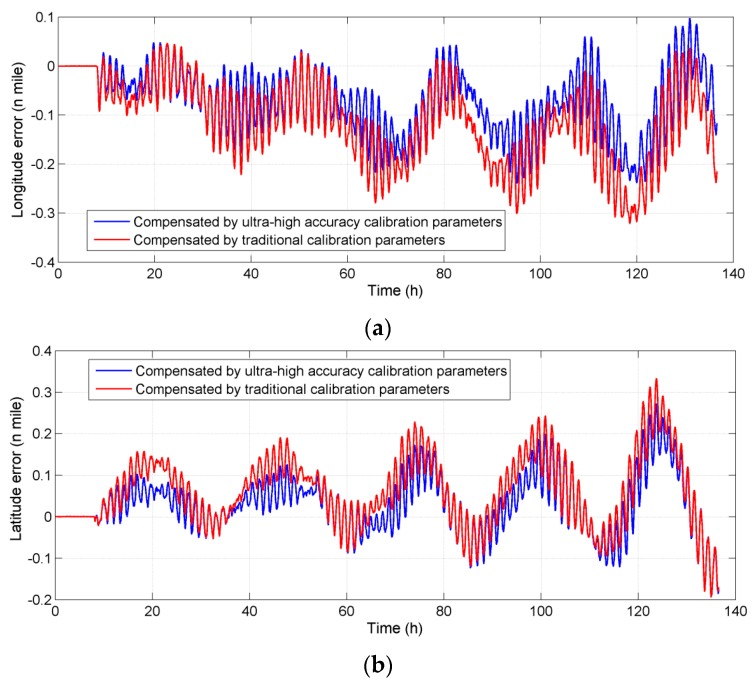
Comparison of the position errors before and after compensation in laboratory tests (**a**) Longitude error; (**b**) Latitude error.

After the calibration test, the navigation test is carried out. The test consists of two parts: initial alignment and pure inertial navigation. It took 8 h for initial alignment and 128 h (about five days) for navigation. During the navigation, the system works in pure inertial navigation modes without any external information for correction, so that all of the errors were accumulated in position errors in order to make a comparison with the effect after calibration and compensation. Raw data was collected during the test and two sets of calibration parameters were used to make an off-line compensation to the raw data. One set of parameters is calibrated by a 30-state Kalman filter, whose accuracy is highest in traditional methods for high accuracy IMU. The other is calibrated by the 51-state Kalman smoother proposed in this paper. The comparison of the position errors of the navigation result before and after compensation is shown in Figure 7. It can be seen that the maximum error of longitude decreased from 0.32 nautical miles (n miles) to 0.24 n miles after compensation, and the maximum error of latitude decreased from 0.27 n miles to 0.25 n miles.

### 4.3. Sailing Test

**Figure 8:**
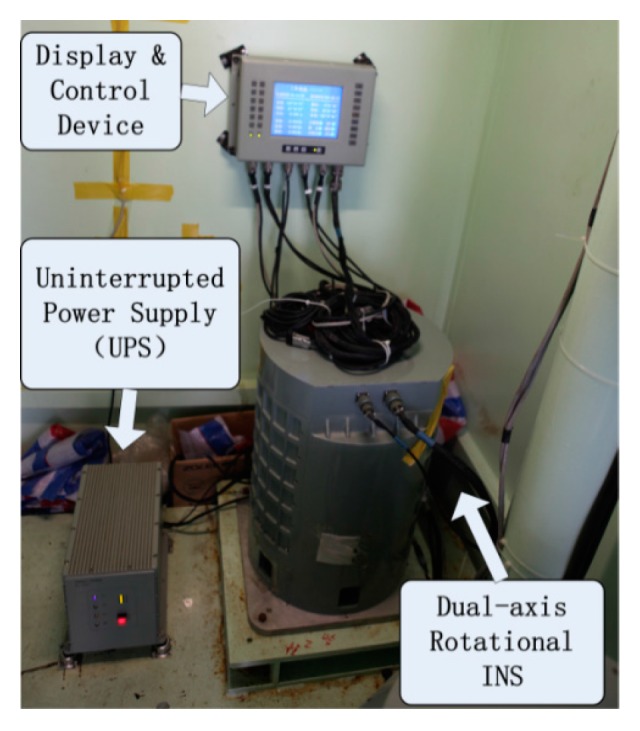
Sailing test arrangement.

In the sailing test, an improved high accuracy marine dual-axis rotational INS with the same accuracy and higher reliability is used. The INS is mounted in a cabin of an experimental ship and the Display and Control Device is mounted on the wall (shown in Figure 8).

**Figure 9:**
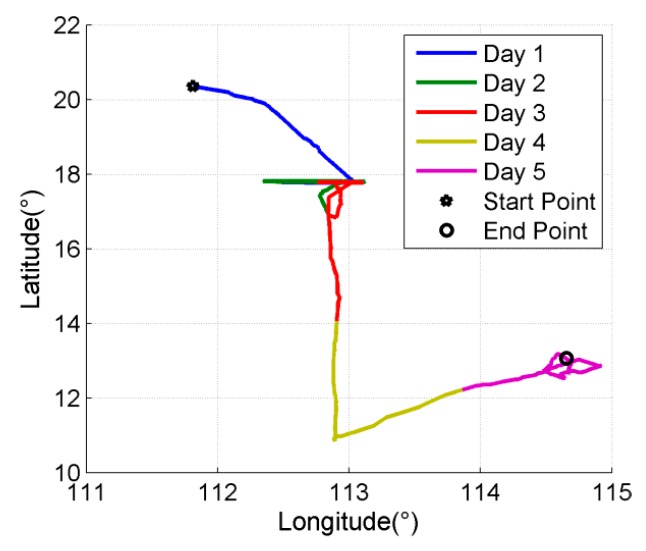
Sailing trajectories.

The sailing test consists 6 h alignment and 108 h (about five days) horizontal-damping navigation at 10~20 degrees north latitude. The velocity information of damping is provided by the on-board log. It has to be pointed out that Schuler oscillation error can be restrained in horizontal-damping mode, but the calibration errors have the same characteristics as in pure inertial navigation. The sailing trajectory is shown in Figure 9.

**Figure 10:**
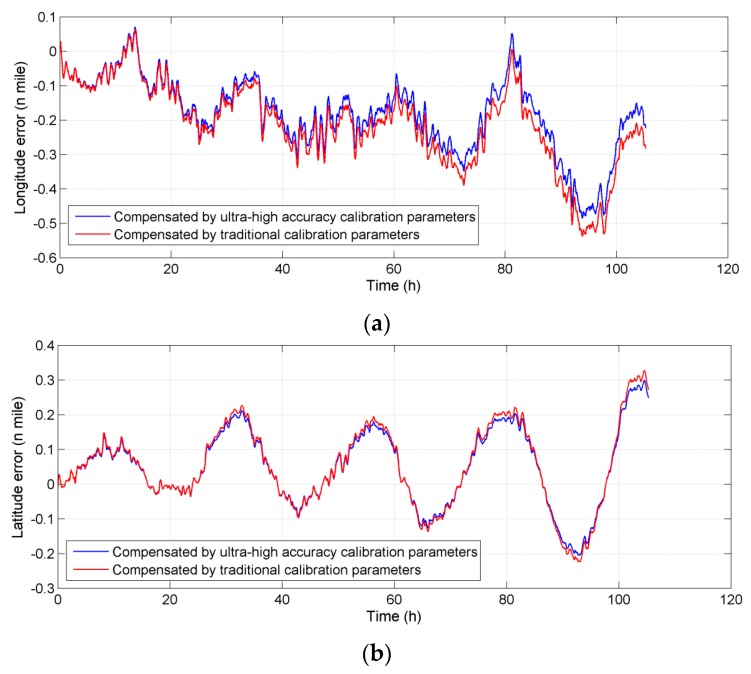
Comparison of the position errors before and after compensation in sailing tests (**a**) Longitude error; (**b**) Latitude error.

Position errors of the navigation results before and after compensation are compared in Figure 10. It can be seen that the maximum error of longitude decreases from 0.53 n miles to 0.46 n miles after compensation, and the maximum error of latitude decreases from 0.32 n miles to 0.28 n miles.

In the laboratory and sailing tests, the position accuracy of dual-axis rotational INS is improved about 8% in the five-day navigation. It proves that the proposed compensation method has an effect on restricting the accumulation error of the system when adopting the existing accuracy level of inertial sensors. For the future ultra-high accuracy atomic gyro INS, the calibration errors discussed in this paper will hold a larger proportion compared with the device error. Assuming that the position accuracy of the atomic gyro INS is much higher than that of the existing INSs, reaching a level of 0.1 n miles/5 d, the accuracy can be improved by at least 20% through calibrating the error parameters introduced in this paper.

## 5. Conclusions

To solve the problem of existing calibration methods and devices not being able to satisfy the accuracy requirement for future ultra-high accuracy inertial sensors, an improved calibration model is established by introducing gyro g-sensitivity errors, accelerometer cross-coupling errors and lever arm errors. A systematic calibration method is proposed based on a Kalman filter and RTS optimal smoothing, and a 51-state equation of systematic calibration filter is established. Compared with the existing calibration methods, a high accuracy calibration model including more error sources and high order small error parameters is established and calibrated in the proposed method by common two- or three-axis turntables. Simulation results show that, for the ultra-high accuracy IMU, the proposed calibration method can realize the calibration of all of the parameters. Laboratory and sailing tests prove that the accumulation errors of the existing highest accuracy INS can be effectively restrained when applying this calibration method; the position accuracy in five-day inertial navigation can be improved by about 8%. Assuming that the position accuracy of the atomic gyro INS can reach a level of 0.1 n miles/5 d, the accuracy can be improved by at least 20% through calibrating the error parameters introduced in this paper. The proposed calibration method for ultra-high accuracy IMUs in this paper has great application potential in future atomic gyro INSs.

## Figures and Tables

**Table 1:** Rotation path of systematic calibration.

Number	Rotation		Attitude after Rotation		
	Rotation Axis	Rotation Angle	*X*-Axis	*Y*-Axis	*Z*-Axis
0	-	-	east	north	upwards
1	outer	+90°	east	upwards	south
2	outer	+180°	east	downwards	north
3	outer	+180°	east	upwards	south
4	inner	+90°	upwards	west	south
5	inner	+180°	downwards	east	south
6	inner	+180°	upwards	west	south
7	outer	+90°	south	west	downwards
8	outer	+180°	north	west	upwards
9	outer	+180°	south	west	downwards
10	outer	+90°	downwards	west	north
11	outer	+90°	north	west	upwards
12	outer	+90°	upwards	west	south
13	inner	+90°	west	downwards	south
14	inner	+90°	downwards	east	south
15	inner	+90°	east	upwards	south
16	outer	+90°	east	south	downwards
17	outer	+90°	east	downwards	north
18	outer	+90°	east	north	upwards

**Table 2:** The simulation result of self-calibration with dual-axis INS.

Calibrated Parameters	Errors before Filter	Errors after Filter
Gyro g-sensitivity error (°/h/g)	0.001	0.0002
Accelerometer nonlinear scale error (μg/g^2^)	300	1.3
Accelerometer cross-coupling error (μg/g^2^)	300	1.5
Accelerometer lever arm errors (cm)	2	0.01

**Table 3:** The calibration result of dual-axis rotational INS.

Parameters	Calibration Result		
Gyro g-sensitivity error (°/h/g)	G_xx_: 0.09 × 10^−5^	G_xy_: 0.25 × 10^−5^	G_xz_: −0.22 × 10^−5^
	G_yx_: 0.35 × 10^−5^	G_yy_: 0.81 × 10^−5^	G_yz_: −0.24 × 10^−5^
	G_zx_: −0.07 × 10^−5^	G_zy_: −0.50 × 10^−5^	G_zz_: −1.02 × 10^−5^
Accelerometer nonlinear scale error (μg/g^2^)	K_axx_: 17.1	K_ayy_: −20.4	K_azz_: 25.2
Accelerometer cross-coupling error (μg/g^2^)	K_axy_: −2.0	K_axz_: −22.6	K_ayx_: 7.8
	K_ayz_: 16.5	K_azx_: −0.5	K_azy_: 1.6
Accelerometer lever arm errors (cm)	r_x_: −4.1	r_y_: 2.2	r_z_: −2.4
